# Equine Dental Pulp Connective Tissue Particles Reduced Lameness in Horses in a Controlled Clinical Trial

**DOI:** 10.3389/fvets.2017.00031

**Published:** 2017-03-10

**Authors:** Alicia L. Bertone, Nathalie A. Reisbig, Allison H. Kilborne, Mari Kaido, Navid Salmanzadeh, Rebecca Lovasz, Joy L. Sizemore, Logan Scheuermann, Rosalind J. Kopp, Lisa J. Zekas, Matthew T. Brokken

**Affiliations:** ^1^Department of Veterinary Clinical Sciences, College of Veterinary Medicine, The Ohio State University, Columbus, OH, USA

**Keywords:** dental pulp, osteoarthritis, lameness, desmitis, tendonitis

## Abstract

**Objective:**

To assess if injection of allogeneic dental pulp tissue particles would improve lameness in horses with naturally occurring osteoarthritis (OA) or soft tissue (ST) injury.

**Design:**

Prospective, randomized, blinded, and controlled clinical trial and client survey assessment.

**Animals:**

Forty lame client-owned horses.

**Procedures:**

Sterile dental pulp, recovered from otherwise healthy foals that perish during dystocia, was processed under good manufacturing processing to produce mechanically manipulated, unexpanded pulp tissue particles containing viable cells surrounded in extracellular matrix. Forty lame client-owned horses with confirmed OA (*n* = 20), or ST injury (desmitis or tendonitis) received a 2 mL intra-articular (*n* = 20 OA) or intra-lesional (*n* = 20) injection of control transport vehicle (*n* = 20) or 10 × 10^6^ dental pulp tissue particles (*n* = 20). Acclimatized horses had baseline measurements performed and were then injected on day 0. Horses were treadmill exercised for 2 weeks, evaluated by clinical parameters, lameness score, edema (score and circumference), pain on flexion (OA) or pressure (ST), and clients’ scores for pain and discomfort before and through 45 days after pulp injection. Twenty horses were available for >2.5-year follow-up.

**Results:**

Pulp-treated horses showed decrease in lameness compared to baseline (*P* < 0.009) or placebo controls (*P* < 0.013) for at least 2 weeks. Client assessments of comfort were improved between before and 45 days after pulp injection (*P* < 0.001). Clinical improvement with ST injury was significantly greater than OA (*P* < 0.001). At >2.5-year follow-up, at least 10 horses were in work.

**Conclusion and clinical relevance:**

Dental pulp tissue particles can be considered as a treatment option for equine lameness due to OA, desmitis, or tendonitis.

## Introduction

Tissue allografts (fresh or frozen) and decellularized extracellular matrix allografts have been used to support osteochondral defects, soft tissue (ST) healing, as well as vascular and ligament replacement for decades (1–3). Allogeneic multipotent mesenchymal stromal/stem cells (MSCs), either isolated or in matrix, potentially offer advantages over autologous tissues for use in regenerative therapies due to lack of patient morbidity at a donor site, well-characterized cells or tissues, accredited manufacturing practices and labeling standards, and practical “off the shelf” access (4–9). A few recent publications in horses have shown measurable differences in clinical response of joints to allogeneic compared to autologous cells (4, 10, 11), however, responses were either not statistically different from controls (11) or were minimal (10) or considered manageable (4). Several human clinical trials have proceeded with allogeneic biologics such as particulated juvenile cartilage allografts^1^ and engineered cartilage allografts^2^ for the treatment of cartilage defects and osteoarthritis (OA). For human cells, successful efficacy for cartilage repair has been demonstrated with allogeneic MSCs and chondrocytes (6, 8, 9, 12–15). Success of these cell-based biologic products has been ascribed to viable progenitor cells that could migrate, engraft, differentiate, and facilitate tissue regeneration (16), but more recently it has been recognized that MSC have anti-inflammatory (17, 18), immunomodulatory (5, 19, 20), and pain-relieving effects (21–23). Several available commercial devices can produce equine patient-side viable cell concentrates of bone marrow,^3^^,^^4^^,^^5^ or adipose tissue,^6^^,^^7^ and are designed for autologous use. However, without culture of the bone marrow or adipose tissue, progenitor and stem cell yields are low (<0.002%) (24, 25). Several studies in horses have specifically supported the use of these cultured processed MSC as therapy for joint and cartilage disease (26–30) as well as ST injury such as tendonitis and desmitis (7, 31, 32). Due to reported success of MSC in these painful lameness conditions in horses, we focused our lameness population to cartilage degeneration and tendonitis/desmitis as target diseases with the primary outcome of lameness relief.

**Figure 1:**
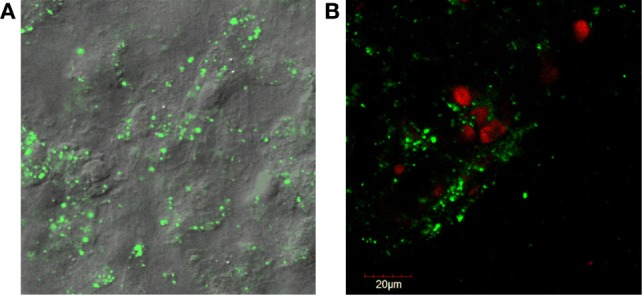
**(A)** Fluorescent confocal microscopy images of dental pulp cell tissue particles showing sheets of cells held together by extracellular matrix. Rounded, raised areas are nuclei and cell borders are indescript due to the attached matrix. The cytoplasm staining green (live) is positive for cell viability (LIVE/DEAD^®1^). **(B)** Counter-staining with ethidium homodimer red (dead) shows that some cells are non-viable as the nuclei stain red. ^1^LIVE/DEAD® Viability/Cytotoxicity Kit, Thermo Fisher Scientific, Waltham, MA, USA.

In humans, dental pulp connective tissue obtained from the base of extracted wisdom teeth has been cryopreserved (33) and progenitor neural, bone, and ligament cells isolated and studied for regenerative potential. Dental pulp from un-erupted tooth buds in humans can be harvested with low morbidity to the donor. Cells can be cryopreserved (33), and subsequently thawed to produce a viable cell for allogeneic or autologous injection. To our knowledge, the study of a dental pulp tissue injectable particulate in horses has not been reported. Dental pulp for the generation of this tissue particulate is obtained from otherwise healthy foals that died during the birthing process (dystocia) using sterile extra-oral harvest techniques by a dedicated surgeon using good tissue practice methods routine for collection of human tissue for allogeneic transplantation. Minimally manipulated tissue processing results in a characterized product, that is available as an “off-the-shelf” cell and matrix product for easy use in practice and avoids some of the limitations with currently reported tissue sources. These limitations for current sources, that are either not a limitation or much reduced limitation for dental pulp, include, for differentiated tissue such as skin and blood, low stem cell yields that often require extraction (34, 35) or mobilization (36), respectively. Umbilical cord and amnion can be contaminated at birth increasing the potential risk of infection in the stem cell harvests. Bone marrow progenitor cell yields are variable due to blood contamination (25), and the procedure can be a risk or painful to the donor (25). Oocytes and other embryonic or fetal sources raise ethical concerns for human tissues and carry the concern of teratogenesis and carcinogenesis (32, 37, 38). Adipose-derived cells require an invasive procedure for harvest and are limited by non-homologous use clauses by regulatory agencies even for autologous use.^8^ Therefore, dental pulp offers a biologic therapy option with benefits of minimally processed tissue, successful cryopreservation, high density of pluripotent cells, avoidance of on the farm processing and product variability, and lack of donor morbidity. Additionally, this promising tissue source of allogeneic stem or progenitor cells has been studied in human tissue and cells (33, 39–48). The high concentration of human progenitor cells in un-erupted dental pulp resulted in robust harvest yields of undifferentiated cells that have been characterized (39). Dental pulp has recently served as a source of human pluripotent cells destined for differentiation into bone (40–42), blood vessels (43, 44), ligament (45), and, uniquely, neural tissue (46–48) resulting in a menu of homologous applications. Human dental pulp has been expanded in culture or immediately cryopreserved and banked (33). To our knowledge, there are no publications on dental pulp as a biologic therapy in horses. Our study will report, in horses, on an injectable mechanically manipulated dental pulp connective tissue particulate^9^ composed of dental pulp cells and extracellular matrix containing growth factors from within the cells and matrix (Figure 1; Tables 1 and 2; see Preliminary Data). These tissue constituents could bring anti-inflammatory and regenerative capabilities to the joint, tendon, or ligament environment (47–50). Dental pulp tissue particles offer a potential alternative or addition to non-steroidal anti-inflammatory drugs or steroids for pain, and with regenerative potential unlike either of those products.

**Table 1:** **Cell viability and receptor expression phenotype immediately post-thaw from equine dental pulp tissue particles as analyzed by flow cytometry (*n* = 25 samples)**.

	Viability	CD45	CD34	CD44	CD90	CD105
Mean (%)	99.05	63.93	79.26	73.94	81.53	63.11
SD	2.50	31.53	22.37	14.62	13.16	17.05

**Table 2:** **Detection and annotation of selected prevalent proteins, found in three samples, in extracellular matrix of equine dental pulp particles by liquid chromatography and mass spectroscopy**.

Protein name	Protein accession numbers	Function summary
Actin	ACT17_DICDI	Various cellular functions such as cytoskeleton structure, cell mobility, chromosome movement, and muscle contraction
Annexin A1	ANXA1_HORSE	Role in the innate immune response as effector of glucocorticoid-mediated responses and regulator of the inflammatory process. Has anti-inflammatory activity. Promotes resolution of inflammation and wound healing
Biglycan	PGS1_HORSE	May be involved in collagen fiber assembly
Collagen 1	CO6A1_HUMAN	Collagen precursor
Collagen 2	CO6A1_MOUSE	Collagen precursor
Collagen 3	CO6A3_HUMAN	Collagen precursor
EMILIN-1	EMIL1_HUMAN	May be responsible for anchoring smooth muscle cells to elastic fibers, and may be involved not only in the formation of the elastic fiber, but also in the processes that regulate vessel assembly. Has cell adhesive capacity
Fibronectin	FINC_HORSE	Bind cell surfaces and various compounds including collagen, fibrin, heparin, DNA, and actin. Fibronectins are involved in cell adhesion, cell motility, opsonization, wound healing, and maintenance of cell shape. Involved in osteoblast compaction through the fibronectin fibrillogenesis cell-mediated matrix assembly process, essential for osteoblast mineralization. Participates in the regulation of type I collagen deposition by osteoblasts
Heat shock protein beta	HSPB1_BOVIN	Involved in stress resistance and actin organization
Hyaluronan and proteoglycan link protein 1	HPLN1_HORSE	Stabilizes the aggregates of proteoglycan monomers with hyaluronic acid in the extracellular cartilage matrix
Myosin	MYH9_CANFA	Cellular myosin that appears to play a role in cytokinesis, cell shape, and specialized functions such as secretion and capping. During cell spreading, plays an important role in cytoskeleton reorganization, focal contacts formation (in the margins but not the central part of spreading cells), and lamellipodial retraction; this function is mechanically antagonized by MYH10
Pentraxin-related protein PTX3	PTX3_MOUSE	Plays a role in the regulation of innate resistance to pathogens, inflammatory reactions, possibly clearance of self-components
Putative actin-25	ACT25_DICDI	Actins are highly conserved proteins that are involved in various types of cell motility and are ubiquitously expressed in all eukaryotic cells. Multiple isoforms are involved in various cellular functions such as cytoskeleton structure, cell mobility, chromosome movement, and muscle contraction
Serpin H1	SERPH_BOVIN	Binds specifically to collagen. Could be involved as a chaperone in the biosynthetic pathway of collagen
Serum albumin	ALBU_HORSE	
60 kDa heat shock protein, mitochondrial	CH60_PONAB	Implicated in mitochondrial protein import and macromolecular assembly. May facilitate the correct folding of imported proteins. May also prevent misfolding and promote the refolding and proper assembly of unfolded polypeptides generated under stress conditions in the mitochondrial matrix
Transforming growth factor-beta-induced protein	BGH3_HUMAN	Plays a role in cell adhesion. May play a role in cell–collagen interactions. Binds to type I, II, and IV collagens
Tubulin	TBA_ENTDO, TBB1_GADMO	Tubulin is the major constituent of microtubules. It binds two moles of GTP, one at an exchangeable site on the beta chain and one at a non-exchangeable site on the alpha chain
Versican core protein	CSPG2_HUMAN, CSPG2_MOUSE	May play a role in intercellular signaling and in connecting cells with the extracellular matrix. May take part in the regulation of cell motility, growth, and differentiation. Binds hyaluronic acid
Vimentin	VIME_BOVIN, VIM4_XENLA, VIMB_CARAU	Vimentins are class-III intermediate filaments found in various non-epithelial cells, especially mesenchymal cells. Vimentin is attached to the nucleus, endoplasmic reticulum, and mitochondria, either laterally or terminally Involved with LARP6 in the stabilization of type I collagen mRNAs for CO1A1 and CO1A2 One of the most prominent phosphoproteins in various cells of mesenchymal origin. Phosphorylation is enhanced during cell division, at which time vimentin filaments are significantly reorganized

In our study, we used this novel viable dental pulp tissue particulate injectable solution (see text footnote 9) that is a minimally manipulated, mechanically processed, unexpanded viable connective tissue targeted for allogeneic use in equine musculoskeletal painful conditions that have reportedly responded to cell-based therapies in horses, specifically joint, tendon, and ligament injury (7, 22, 26–31). We predicted that dental pulp, minimally and mechanically processed, used as an injectable treatment for painful ST injury (tendonitis or desmitis) or OA, would affect those events associated with tissue injury (pain and inflammation) and result in less lameness. Growth factors within or stimulated by the dental pulp tissue treatment may also have the potential to prevent tissue degeneration and be disease-modifying (48–50).

We aimed to determine if injection of equine allogeneic dental pulp tissue particles into naturally occurring equine cases of inflamed injured tendon or ligament or OA joints would alter lameness and other parameters of pain, produce inflammation at the injection site, or show disease-modifying criteria as measured by joint biomarkers. We targeted the painful conditions of OA, tendonitis, or desmitis (ST injury) and used a prospective blinded randomized controlled clinical trial under tight environment control and with quantitative outcomes as our design. Our hypothesis was that injection of test article (see text footnote 9) would have significant improvements in outcomes related to lameness (scores and force plate parameters), pain (joint flexion, lesion pressure), or inflammation (circumference, scores) as compared to an injection of a similar volume of the carrier vehicle without dental pulp particles.^10^ Based on prior publications it was anticipated that, if there was an influence, this could be proven within 2 weeks (22). Additionally and simultaneously, we performed a client assessment of comfort using a validated survey in a prospective manner such that baseline scores allowed each horse to serve as its own control. The impact of this work would be to, first, potentially prove that dental pulp tissue particles can reduce lameness and pain in horses affected with one of our most common diseases causing lameness and, second, that client assessment of improvement in comfort and satisfaction will support this finding. Additionally, long-term surveys could document that previously injected horses that could not perform at the time of enrollment could return to work without associated adverse life-threatening events or worsening of their condition.

## Preliminary Data

### Equine Dental Pulp Tissue Particle Cellular Characterization

The dental pulp connective tissue’s minimal processing results in particles of cells and ECM less than 100 μm in size with a hydrodynamic average diameter of 20 μm. Pulp tissue particles are mostly ECM and cell(s) bound together but also occasional ECM or cell(s) alone. Many of the larger particles (>50 μm) contain multiple cells. There are 10 × 10^6^ ECM/cell(s) particles in each 2 cc injection. Cellular characterization was performed on cryopreserved vials of dental pulp connective tissue particulate suspension from 25 different tissue donations, thawed from the −80°C quarantine freezer and placed in a bio-laminar flow hood. Seven culture test tubes were labeled as follows: (1) unlabeled, (2) CD34-FITC (11-0341-82^11^), (3) CD45-PE [12-0450-82 (see text footnote 12)], (4) CD44-FITC [12-0441-82 (see text footnote 12)], (5) CD90-PE [12-0909-42 (see text footnote 12)], (6) CD105-PE [12-1051-82 (see text footnote 12)], and (7) propidium iodide [00-6990-50 (see text footnote 12)]. The thawed tissue suspension was washed and re-suspended in 2.10 mL of transport solution. Tissue suspension (300 μL) was pipetted into each of the labeled culture tubes to which 20 μL of the appropriate antibody was added. No antibodies were added to the unlabeled cell population, but the same steps were completed as for samples with antibodies. Cells were placed at 8°C in the dark for 30 min, and tubes were vortexed at the 15 min mark. After 30 min, sterile transport solution was added to each tube and centrifuged at 1,800 RPM for 7 min at room temperature. After decanting, the cells were re-suspended in 500 μL of sterile transport media. Aluminum foil was placed around the tubes to protect them from light. The foiled tubes were transported in a cooler with an ice pack and brought to the flow cytometry lab at the university to have the cells analyzed.^12^ Cells were >99% viable post-thaw (Table 1). Confocal live/dead fluorescent microscopy assay (see text footnote 10) of the dental pulp tissue particles also showed a majority of live cells that were surrounded by a varying amount of extracellular matrix in some cases maintaining the cells as layered sheets (Figure 1). In conclusion, equine dental pulp cells highly expressed progenitor and stem cells markers, from both hematopoietic (CD45+ and CD34+) progenitor lines, as well as mesenchymal (CD44, CD90, and CD105) progenitor lines, indicating a primordial cell capable of multi-lineage differentiation.

### Equine Dental Tissue Pulp Particle Extracellular Matrix Protein Characterization

The extracellular matrix was analyzed for protein composition using liquid chromatography and mass spectroscopy^13^ and the annotated proteins with the greatest prevalence were summarized from positive hits for 253 proteins in the extracellular matrix. The most prevalent proteins were annotated and categorized by function according to the National Center for Biotechnology Information^14^ and included many proteins with anti-inflammatory activity (Annexin A1), structural elastic, and adhesion activities in MSC (vimentin, versican core protein, tubulin, myosin, fibronectin, EMILIN-1, collagen 3), proteins of stress resistance, fiber assembly, and compressibility (biglycan, heat shock protein, hyaluronan, proteoglycan link protein, serpin H1), growth factors (transforming growth factor-beta-induced protein), and innate pathogen resistance (pentraxin-related protein PTX3) (Table 2). In conclusion, many of these proteins could serve a function as anti-inflammatory and/or regenerative in inflamed and healing tissue.

### Pilot Equine Studies in Normal Horses

**Figure 2:**
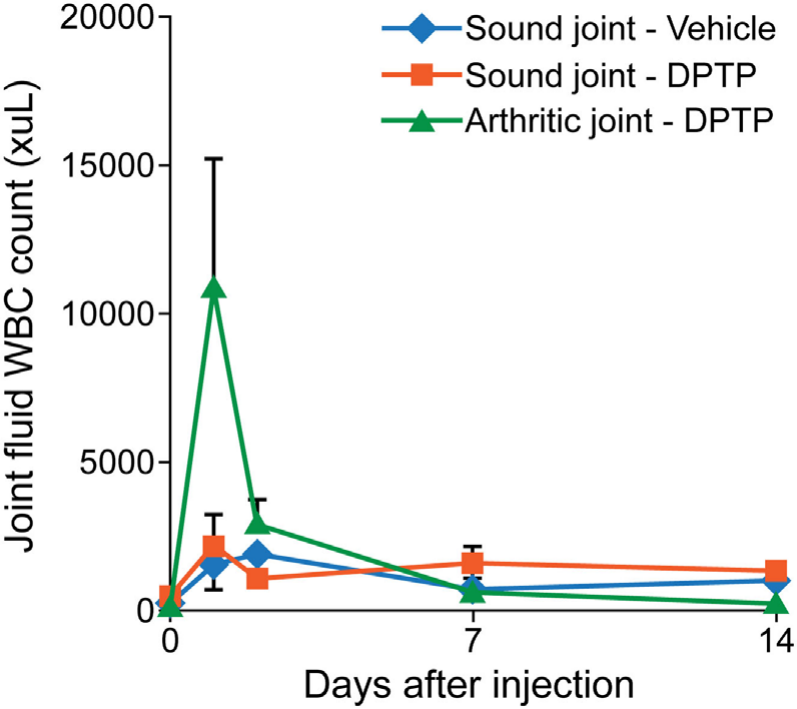
**Graph of total nucleated cell counts from synovial fluid of sound horses’ normal joints injected with transport control vehicle (blue) or dental pulp tissue particles (red) or lame horses’ osteoarthritic joints injected with dental pulp tissue particles (green)**. Joints with osteoarthritis had a greater nucleated cell count than normal joints at day 1 after injection.

Three adult, university owned, research horses with no musculoskeletal abnormalities in the bilateral forelimb fetlock joints, at day 0, had one randomly chosen forelimb fetlock joint injected with 1 × 10^6^, 5 × 10^6^, or 10 × 10^6^ equine dental pulp tissue particles as 1 mL volume in transport vehicle, and the contralateral fetlock joint was injected with the equivalent volume (1 mL) of transport vehicle alone. Joint fluid from both joints were obtained at days 0, 1, 2, 7, and 14, and analyzed for total protein concentrations, white blood cell (WBC) count, and neutrophils (%). Pain and swelling of each joint was assessed at days 0, 1, 2, 3, 7, and 14, by measuring joint circumference (centimeters), joint swelling score (0–4), pain-free range of joint motion (degrees), and pain on flexion score (0–4) as described in more detail in the main study. Lameness was assessed at days 0, 3, 7, and 14, by using a lameness grading scale from 0 to 5, before and after flexion of the injected joints. Venous blood was drawn at days 0 and 14 and analyzed by complete blood count (CBC) and chemistry panel, and the body weight was measured at days 0, 7, and 14. This dose escalation study demonstrated similar synovial fluid inflammation of the vehicle alone and the 1 × 10^6^ or 5 × 10^6^/mL dose (Figure 2). Based on these findings, the dose of 5 × 10^6^/mL was selected for use in subsequent studies to maximize the dose along with minimizing the inflammation.

### Pilot Preclinical OA Study

**Figure 3:**
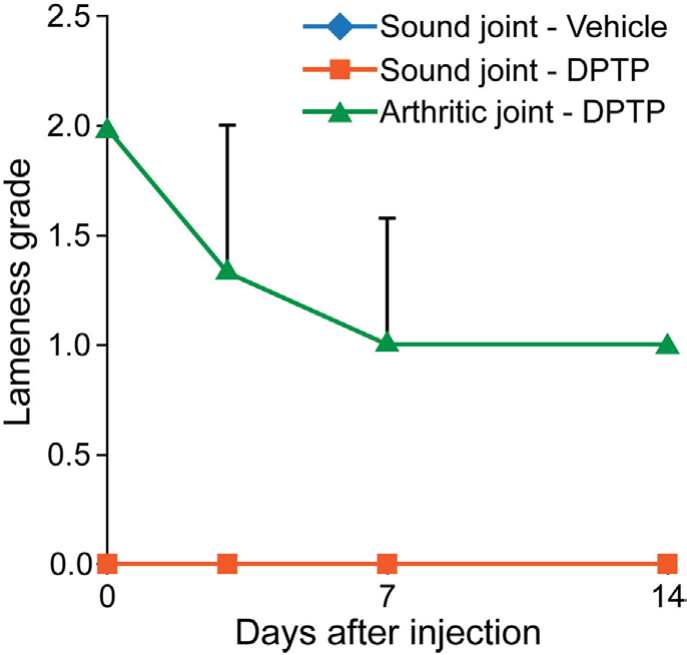
**Graph of lameness scores of sound horses’ normal joints injected with transport control vehicle (blue) or dental pulp tissue particles (red) or lame horses’ osteoarthritic joints injected with dental pulp tissue particles (green)**. Lameness in horses with osteoarthritis significantly improved (*P* < 0.05) from baseline to day 14.

Three adult, university owned, research horses, diagnosed with OA in a radiocarpal, midcarpal joint, or hindlimb fetlock joint, were used in this pilot study. Inclusion criteria included a score >2 for lameness and radiograph within 30 days demonstrating signs of OA including osteophyte formation, joint space irregularity or narrowing. At day 0, the affected joint was injected with 5 × 10^6^ allogeneic dental pulp tissue particles in 1 mL of transport vehicle. Other parameters measured were similar to the dose escalation study. The results showed an increase in synovial fluid WBC count on days 1 and 2 after injection, greater than in normal horses (Figure 2). No lameness was detected in limbs injected with vehicle or 5 × 10^6^ dose in normal sound horses, and lameness decreased (*P* < 0.001) in OA horses between days 0 and 14 (Figure 3). Joint swelling scores, pain-free range of joint motion, and pain on flexion scores were not significantly different among groups or any time point. Swelling was localized to the joint and modest in amount. There was no significant difference in any blood parameters measured among groups or between time points (days 0 and 14).

## Materials and Methods

### Prospective, Randomized, Blinded, and Placebo-Controlled Clinical Trial Design

Forty lame horses [American Association of Equine Practitioners (AAEP) lameness grades 2–5 (22); age range 2–18 years] with OA (*n* = 20), or with ST injury, either desmitis or tendonitis (*n* = 20), were assigned, by use of an online random number generator,^15^ into either a treatment group (*n* = 20) or control group (*n* = 20) based on serial enrollment within OA or ST diseases. At day 0, the horses in treatment and control groups received an injection of dental pulp tissue particles (see text footnote 9) or control sterile buffered isotonic saline transport solution (see text footnote 11), respectively. All horses were evaluated by physical examination’s (day 0, day 7, day 14), blood analysis (screening, day 2, day 14) and joint synovial fluid analysis (OA; day 0, day 14), use of subjective lameness grade scores (AAEP 0–5; baseline, day 7, day 14), as well as measuring or grading inflammation parameters {edema, limb circumference [baseline, days 1 (edema only), 2, 4, 7, 10, 14]}, pain score on palpation (ST), goniometric range of motion (OA), lameness pain score on pressure (ST; baseline, days 2, 4, 7, 10, 14), and pain score on lameness flexion (OA; day 0, day 7, day 14). Ultrasound (baseline, day 7, day 14) and radiography (baseline, day 14) were also performed. Horses were confined to a 12′ × 12′ boxstall and exercised twice weekly on a treadmill for the duration of the study. Owners were blinded as to the assignment for the duration of this study. After study termination at day 14, owners of horses in the control group were offered the option for their horses to receive an injection of dental pulp tissue particles following the same protocol as the treatment group.

### Prospective Client Assessment Study Design

All clients with horses enrolled in the prospective, randomized, blinded, and placebo-controlled clinical trial were simultaneously enrolled in a client assessment study of their horses’ status using a survey that corresponded to lameness and pain in our prior work (22).^16^ Clients enrolled at the start of the study prospectively agreed to complete all forms at the times on the protocol, which was before assignment of their horse into the clinical trial and at days 21 and 45 after injection. To complete the survey, the horse was to be observed in several different activities over a day or so until the client was comfortable that the scores reflected the condition of the horse. These assessments were direct visual observation by the client and data recorded on our survey questionnaire form, signed and dated by the client. Clients performed visual assessments requested by the questionnaire on their horse in the horse’s home environment before transportation to the study site for enrollment. Owner’s committed in the signed consent form that their horse’s environment would remain the same until after the final questionnaire. Their first post-injection assessment would occur shortly after the horse returned home and another in 3–4 weeks after the horse returned home. Clients were instructed to return the horse to the level of activity they were on prior to enrollment at their baseline assessment, only varied if recommended by the author (Alicia L. Bertone). Both before the enrollment and at enrollment, each client had a discussion session with the author (Alicia L. Bertone) to go over the forms and answer any questions the client may have had in interpreting the questions in the survey. Seven categories were graded by the client on a Likert scale ranging from 0 to 10: degree of lameness (0 = sound; 10 = won’t bear weight), comfort at rest in stall (0 = comfort; 10 = discomfort), comfort at turnout (0 = comfort; 10 = discomfort), general attitude (0 = bright, alert, and interactive; 10 = dull, signs of depression, and non-interactive), appetite (0 = eats all food and looks for more; 10 = will not eat), body condition (0 = excellent; 10 = poor), and hair condition (0 = excellent; 10 = poor). All client assessments were blinded at baseline assessment and unblinded at follow-up because all horses received dental pulp tissue particle injection at either day 0 or after day 14 resulting in a lower level of evidence than the prospective randomized blinded placebo-controlled clinical trial. This client assessment study, however, did meet several requirements that supported a level of evidence for these data. Clients were instructed in the use of the survey and signed and dated all forms as part of the good clinical practice standards used for this trial. All horses were on the same environmental protocol and outcome assessments creating a tightly controlled group of lame horses that had met strict inclusion criteria for their pain and lameness along with a confirmed diagnosis with the result of a tight data set for the client’s assessments. Horses were randomly assigned to the study conditions and conditions at home were maintained for both baseline and post-injection assessments. All clients (100%) were enrolled to complete this validated questionnaire as part of the study at defined times, and all enrollments were completed within a 6-month time frame. For this phase, each horse served as its own control. Additionally, the clients were queried by the author (Alicia L. Bertone) for adverse events including worsening of lameness and any events were recorded on the adverse event form, signed, and dated. These same clients were contacted by phone or email 2.5–3 years after injection and answered the same questionnaire.

### Inclusion Criteria

At admission horses had to have a diagnosis of lameness due to clinical evidence of OA, desmitis, or tendonitis. Diagnosis of OA, desmitis, or tendonitis was determined within 30 days prior to or at arrival for enrollment by use of lameness, radiographic examination, ultrasound examination and, when indicated, diagnostic anesthesia to confirm the location of the lameness. Clinical criteria for OA were defined as lameness, joint swelling, pain or reduction in joint flexion in the presence of radiographic osteophytes, subchondral bone sclerosis, or narrowing of the joint space. Clinical criteria for desmitis were defined to include lameness with ultrasonographic changes indicating areas of enlargement, a hypoechoic pattern (indicative of edema), or disruption of fiber pattern in the suspensory ligament. Tendonitis maintained the same definition as desmitis but signs in conjunction with lameness could be the aforementioned ultrasound changes for desmitis or a visual lateral profile of superficial digital flexor tendon (SDF) or deep digital flexor tendon (DDF) swelling or pain on pressure of the SDF or DDF. For study purposes, all lameness scores on initial evaluation had to fall between 2 and 5 on the AAEP scale and be at least 4 weeks in duration. Exclusion criteria included presence of an infection, unstable fracture, or to have received tendon, ligament or joint injection (within 2 months), or surgery (within 6 months). All procedures were approved by the Institutional Animal Care and Use Committee. Informed consent was obtained from owners of horses before enrollment in the study. All horses had jugular venous blood submitted for CBC before and at the termination of the study.

### Equine Dental Pulp Tissue Processing

These dental particle allografts were mechanically dissociated with disposable sterile tissue grinders, counted and re-suspended at a concentration of 5 × 10^6^ cell particles/mL, cryopreserved, and stored in liquid nitrogen for long-term storage. For the dental pulp tissue particles used in this study, tissue was thawed, washed and centrifuged, and re-suspended at a concentration of 5 × 10^6^/mL in a sterile isotonic buffered saline solution (see text footnote 11), ready to inject. Prior to cryopreservation and prior to injection, dental pulp tissue particles were tested for transmissible diseases including microbial, fungal, and viral as per American Association of Tissue Bank guidelines.^17^ Post-thaw viability was confirmed with flow cytometry (propidium iodide). Particles diluted to aforementioned dose concentrations, were shipped overnight to the clinician for immediate injection or storage at 8°C for up to 10 days.

### Treatment

Injections of either dental pulp tissue particles suspended in sterile buffered isotonic saline solution (5 × 10^6^ particles/mL) or the sterile buffered isotonic saline solution without pulp particles (placebo control) were delivered on day 0 as 2.5 mL samples to the study site and placed in a dedicated, locked refrigerator, in a coded vial for blinding, until used. After day −1 and before injection, all baseline parameters were recorded. At day 0 in OA horses, joint fluid aspiration followed by intra-articular injection of dental pulp tissue particles (treated group; 2 mL of transport solution containing 10 × 10^6^ pulp tissue particles) or transport solution (control group; 2 mL) was performed by one investigator (Alicia L. Bertone), who was kept unaware of treatment assignments. Aspiration of, and injection into, the joints was performed by passing a needle percutaneously into the joint after clipping and aseptic preparation of the injection site. Joint fluid aspiration confirmed needle placement in the joint and up to 5.0 mL was aspirated prior to the injection. The fluid withdrawn was submitted for standard synovial fluid analysis and cytology.^18^ Remaining synovial fluid was frozen at −20°C for ELISA analysis of biomarkers interleukin-1 receptor antagonist (IL-1ra), interleukin-1β (IL-1β), interleukin 6 (IL-6), and interleukin 10 (IL-10).^19^ Bandages were not applied after the joint aspiration and injection. At day 14, joint fluid aspiration was performed again in all OA horses. For control OA horses, aspiration was followed by intra-articular injection of dental pulp tissue particles after day 14, if elected by the client, following the same protocol as for the treatment group.

At day 0 in ST horses, the epicenter of the ST lesion was located with ultrasound, marked with a skin staple, and injected percutaneously, after an aseptic preparation as described above for joint injection, with 2 mL of transport solution containing 10 × 10^6^ pulp tissue particles (treated group) or 2 mL transport solution (placebo-control group). All injections were performed by one investigator (Alicia L. Bertone).

### Exercise

Horses were exercised twice per week on a high-speed equine treadmill^20^ for 10 days of acclimation and 2 weeks of the study with the following regimen: walking (7.2 km/h) for 10 min, followed by trotting (14.4 km/h) for 10 min, and walking (7.2 km/h) for 10 min. If horses were or became lame at the walk on the treadmill, they were not trotted and were exercised at the walk only or hand-walked. Throughout the experiment, the horses were housed individually in stalls in a temperature-controlled environment, fed a commercial grain mixture twice daily, and provided access to hay and water *ad libitum*. No medications or supplements were given for at least 1 week prior to and during the 2-week study period.

### Lameness Examination

Evaluation of lameness was performed on the basis of a modification of the AAEP lameness scale (22) on enrollment and at days 0, 7, and 14 by one investigator (Alicia L. Bertone) unaware of group assignments. In a 30-m-long lameness examination area with a smooth, flat paved surface, lameness was graded with the horse being walked in one trip (30 ft back and forth—60 ft total), trotted in two trips with walking on the turns (to the left on the first trip and to the right on the second trip) and trotted in one trip after joint flexion in OA horses [30 s (lower limb) or 60 s (upper limb)] or after tendon/ligament pressure in ST horses (30 s). Lameness grades were as follows: 0 = not lame at the walk and trot, 1 = not lame at the walk and intermittently lame at the trot, 2 = not lame at the walk and consistently lame at the trot under special circumstances of lunging, 3 = not lame at the walk and consistently lame at the trot on straight line, 3.5 = obviously lame at the trot on a straight line and lame at the walk on the turns, 4 = lame at the walk and trot on a straight line, and 5 = partially or fully non-weight bearing at the walk. Additionally, using a scale of 1–9, horses were evaluated for severity to be added to their lameness score as a decimal place (e.g., a horse consistently, but minimally lame at the trot would be graded a 3.1 and a horse markedly and obviously lame at the trot, but not the walk, would be graded a 3.9). Flexion tests (OA) or palpation pain pressure tests (ST) were performed and a lameness grade reflecting the response to the correlating stress test was recorded. A lameness index was calculated as the sum of scores for baseline lameness (0–5), the severity score (0.1–0.9), and the scores for lameness in response to flexion test or palpation/pressure test [i.e., a lameness grade 3 with a severity grade 3 was recorded as a 3.3 and added to the score after joint flexion (e.g., 4) therefore this lameness index would be 7.3 (3 + 0.3 + 4)]. These lameness indices were then evaluated similarly to previous lameness data and reflected pain. In addition, the frequency of change in lameness was calculated by comparing the initial lameness score to the final day 14 lameness score for each horse and recorded as improved (score decreased), no change (score the same), or worsened (score increased).

### Kinetic Gait Analysis

An examination aisle (3 m × 20 m) with a non-slip mat covered force plate^21^ and computer analysis system (see text footnote 21) was used for kinetic gait analysis of each horse at baseline, days 7 and 14 (22). Force plate data were obtained following the subjective lameness scoring. Data sampling rate was 500 Hz, and data were filtered by a stop-band frequency of 80 Hz. Data collection included five valid strikes (passes) of the lame and contralateral limb for each time point. A valid measurement was defined as a passage by the horse over the force plate during which the hoof of the limb of interest fully contacted the surface of the plate and the gait velocity was within the range of 2.5–3.5 m/s. Should horses become too lame to keep the 2.5–3.5 m/s pace, their velocity ranges were reduced, recorded, and maintained at the slower velocity for the entire study for that horse. Vertical force peak (VFP; *n*/kg), vertical force impulse (VFI), and velocity were recorded (22). To adequately compare data that included forelimbs and hindlimbs, each horse was used as its own control and values were expressed as a ratio to baseline and as an asymmetry index (AI). AI between lame and contralateral limbs were expressed as a percent (21–23) and calculated as follows: $\left(\mathrm{VFP}{\left(\mathrm{VFI}\right)}_{\mathrm{Contralateral}}-\mathrm{VFP}{\left(\mathrm{VFI}\right)}_{\mathrm{Lame}}\right)÷\left(\left[\mathrm{VFP}{\left(\mathrm{VFI}\right)}_{\mathrm{Contralateral}}+\mathrm{VFP}{\left(\mathrm{VFI}\right)}_{\mathrm{Lame}}\right]÷2\right)\times 100$ (22).

### Imaging Examinations

#### Radiology

Standing cranio-caudal and lateral radiographs of the affected joint were taken at screening, and day 14 to meet inclusion criteria or document a bone or joint adverse reaction. All radiographic images were assessed semi-quantitatively by an American College of Veterinary Radiology diplomate (Lisa J. Zekas), unaware of group assignments, and scored (0 = none, 1 = minimum, 2 = mild, 3 = moderate, or 4 = marked) for osteophyte formation, joint narrowing, and sclerosis and irregularity of subchondral bone as well as the presence of subchondral bone cysts.

#### Ultrasound

**Figure 4:**
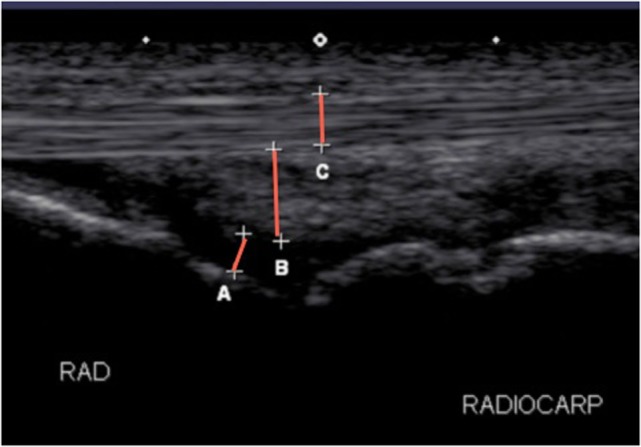
**Longitudinal ultrasonographic image of a radiocarpal joint detailing the measurements performed in the study for joints**. (A) Joint fluid, (B) joint capsule, and (C) tendon and total depth were measured at the same site from skin to bone.

Ultrasound examination of the standing sedated horse in cross and longitudinal section were similarly taken at screening, and days 7 and 14 to confirm the diagnosis in tendonitis or desmitis horses as well as for identification of the amount and location of inflammation in all horses and identification of short-term local adverse reactions. For flexor tendonitis or suspensory desmitis, a complete standard tendon/ligament ultrasound was performed, including longitudinal and cross-sectional images. For assessment of swelling, a central longitudinal ultrasound was selected for the joint, tendon or ligament affected and subsequently measured (centimeters) for total depth (bone to skin), synovial fluid depth in joints only (bone to joint capsule margin), tendon thickness, and subcutaneous tissue thickness (Figure 4). To obtain consistent depth measurements of the joint capsule, the measurements at all time points began at a constant depth below the bones and constant width between the bones. The site of the ultrasound on the limb on day 0 was marked with a stainless steel staple and images were recorded for later analysis. The measurements were carried out through eFilm Workstation^22^ by two investigators blinded to treatment (Lisa J. Zekas and Logan Scheuermann).

### Statistical Analyses

All statistical analyses were performed by use of commercially available statistical software programs.^23^^,^^24^ The effects of injections on lameness scores, kinetic gait measurements, signs of inflammation (edema, limb circumference), goniometric range of motion, and pain on manipulation (flexion or pressure), and ultrasound measurements were analyzed by use of 2-factor repeated-measures ANOVA (factor 1 = treatment, factor 2 = time). Explanatory variables included horse, gender, limb (forelimb or hind limb; right or left), time points, and treatment (carrier vehicle or dental pulp tissue particles). Each horse was treated as a random variable, and repeated measures (time points) were considered to be nested within horse. For kinetic analyses, coefficients of variation (CV) were calculated for VFP, AI-VFP by dividing SD by the mean VFP. To test whether VFP was significantly altered by the classification of disease, the original data set was analyzed in total and divided into three disease groups (OA, desmitis, or tendonitis). Since there was no statistical difference between the ST injuries for desmitis and tendonitis, these were also combined for additional analyses. For each disease subset, repeated measure analysis was repeated as above.

Lameness parameters were also evaluated by analysis of the frequency of horses responding or not responding to treatment. Horses were categorized as responders if the changes in subjective lameness grades were decreased from day 0. Horses were categorized as non-responders if the lameness grade increased from day 0. The categories of responder or non-responder for lameness grade (days 7 and 14) were compared between the explanatory variables for frequencies by χ^2^ test. Lameness scores were also correlated to CV-VFP and AI-VFP using Pearson’s correlation coefficient.

Values of *P* ≤ 0.05 were considered significant for all analyses. In prior published work, lameness score, AI-VFP, and client scores had significant differences (*P* < 0.05) between treated and control groups in OA horses that indicated statistically significant improvement is lameness (22). Based on the means and SDs from that study, accepting a power of 0.8 and alpha error of 0.05, to detect, *a priori*, a difference in lameness score of 1, a difference in AI-VFP of 30%, and a difference in client score of 1, a sample size of three per group is sufficient^25^. Our study should be well powered with an *n* = 10 horses per category for our primary outcome variables.

## Results

### Horses

Forty client-owned horses (age range 2–17 years; mean 12.6 years; mean body weight 537 kg; 25 geldings, 1 stallion and 14 mares), meeting inclusion and exclusion criteria, and diagnosed with painful OA (*n* = 20), suspensory desmitis (*n* = 14), or tendonitis (*n* = 6) of the distal limb were enrolled in the study between December 2013 and June 2014. Breeds represented were Warmblood Sport Horses (15), Quarter Horses (13), Thoroughbreds (6), Arabians (2), Standardbred, Morgan, Missouri Fox Trotter, and American Paint Horse at (1) each. Front limb lameness and hind limb lameness were not different between the treated (*n* = 13, *n* = 7, respectively) and control horses (*n* = 10, *n* = 10, respectively). There were no significant differences in right or left lame limb, breed distribution, age, gender, or body weight, between the treatment and control groups. All horses completed the study. No complications occurred in the preparation or injections of dental pulp tissue particles or control solution.

### Physical Examination

The parameters of rectal temperature, pulse rate, and respiratory rate were out of reference ranges^26^ 24 of 363 times and were deemed acceptable (Alicia L. Bertone). CBC parameters were within references ranges (see text footnote 17) and deemed acceptable in all horses. There were no significant differences in the physical examination parameters or CBC parameters between groups or across time.

### Synovial Fluid Parameters

**Figure 5:**
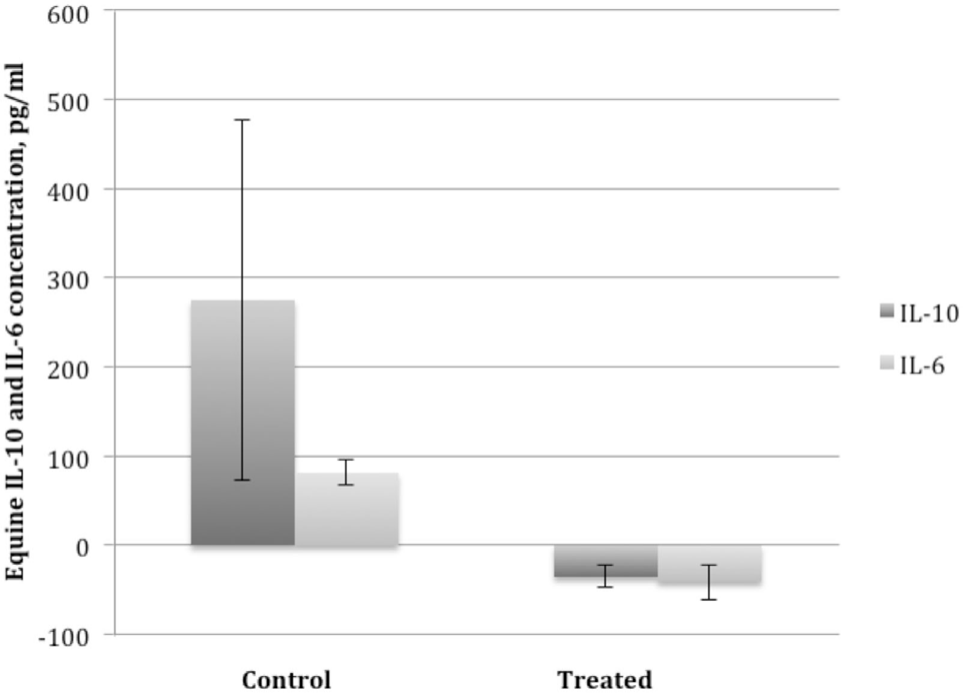
**Mean ± SEM of the change in interleukin 10 (IL-10) and interleukin 6 (IL-6) concentration in synovial fluid of horses with osteoarthritis from before administration of dental pulp tissue particles (treated) or transport solution (control) on day 0, prior to injection (left bar) to day 14 after administration (right bar)**. Treated horses had a significant decrease and control horses a significant increase in IL-10 and IL-6 concentrations compared to baseline (*P* < 0.03).

Synovial fluid was of sufficient volume for analysis at days 0 (10 of 20 samples) and 14 (9 of 20 samples) in horses with OA. Synovial fluid WBC counts at day 0 and day 14 were within normal reference ranges (see text footnote 17) (<1,000 total cells/μL) with counts >1,000 cells/μL rare (two samples) and only seen with blood contamination. No significant difference in synovial fluid analysis was noted between treated- and control-OA horses. Synovial fluid from dental pulp tissue particle-treated joints had significantly lower concentrations of IL-6 (*P* < 0.03) and IL-10 (*P* < 0.03) on day 14 compared to day 0 while control joints had a significantly greater concentration of IL-6 and IL-10 on day 14 (*P* < 0.03) (Figure 5). There was no significant difference in IL-1ra or IL-1β, or their ratio in control or treated groups at any time point.

### Edema/Swelling

Edema scores had a significant time effect (*P* < 3.3E−30) and were greater than baseline in injected horses in both groups up to and including day 10. Treated horses had greater edema score than control horses at days 2, 4, and 7 (*P* < 0.001) but both groups ranged from 0 to 3. Limb circumference data were expressed as a change from baseline (day 0) and showed no significant difference between groups but a significant effect of time (*P* < 0.04). The greatest increase in circumference was at day 2, then showed a decrease in limb circumference over time (Table 3).

**Table 3:** **Parameters measured (mean ± SD) to assess inflammation and pain after injection of dental pulp tissue particles or carrier vehicle in horses with distal limb lameness due to osteoarthritis (OA) or soft tissue (ST) injury of tendonitis or suspensory desmitis**.

Parameter	Disease	Group	Day 0	Day 2	Day 4	Day 7	Day 10	Day 14
Edema 0–4	All	C	0.09 ± 0.29	1.05 ± 0.7^+^^,^*	1.05 ± 0.8*	0.86 ± 0.8*	0.45 ± 0.67	0.23 ± 0.4
		Tx	0 ± 0	1.55 ± 0.9^+^^,^*	1.6 ± 0.88^+^^,^*	1.5 ± 0.83*	0.65 ± 0.8	0.3 ± 0.66
Circumference (Δ mm from day 0)	All	C^+^ (*P*<0.002)		0.54 ± 0.5	0.62 ± 0.6	0.19 ± 0.5	−0.09 ± 1.3	0.63 ± 2.1
		Tx^+^ (*P*<0.002)		0.87 ± 0.8	0.64 ± 0.69	0.37 ± 0.65	0.24 ± 0.54	0.12 ± 0.45
	ST^#^ (*P*<0.004)	C^+^ (*P*<0.004)		0.71 ± 0.6	0.6 ± 0.5	0.35 ± 0.36	−0.11 ± 1.6	0.26 ± 0.24
		Tx^+^ (*P*<0.004)		0.73 ± 0.9	0.73 ± 0.9	0.58 ± 0.66	0.23 ± 0.56	0.12 ± 0.38
Pain score (0–4)	All	C	0.8 ± 0.9	0.4 ± 1.0	0.4 ± 1.0	0.9 ± 0.9	0.3 ± 0.9	0.6 ± 0.9
		Tx	1.4 ± 1.7	0.6 ± 1.1	0.5 ± 1.1	1.1 ± 1.5	0.4 ± 0.8	1.1 ± 1.6
	OA (to flexion)	C	1.4 ± 1.7			1.6 ± 1.7		1.0 ± 1.1
		Tx	2.3 ± 1.9			2.0 ± 1.8		1.9 ± 1.9
	ST (to pressure)	C	0.3 ± 0.9	0.4 ± 1.0	0.4 ± 1.0	0.3 ± 0.9	0.3 ± 0.9	0.3 ± 0.9
		Tx	0.5 ± 1.1	0.6 ± 1.1	0.5 ± 1.1	0.4 ± 0.8	0.4 ± 0.8	0.4 ± 0.8
Joint range of motion (degrees)	OA	C	118.6 ± 54.1	118 ± 61.2	122.7 ± 55.3	118.6 ± 60.4	118.5 ± 59.5	116.4 ± 60.2
		Tx	119.6 ± 57.7	112.2 ± 58.3	118.4 ± 59.2	115.6 ± 56.5	117 ± 58.4	117.4 ± 58.1

*Differs between control and treated group (*P* ≤ 0.05).

*^+^Differs from baseline within group (*P* ≤ 0.05)*.

*^#^Differs across time within ST group (*P* ≤ 0.05)*.

### Pain Scores and Pain-Free Range of Motion in OA Horses

Scores for pain on flexion of OA horses and pain on pressure for all ST horses showed no significant difference between groups or across time. A significant increase in pain induced lameness scores (on pressure test) specifically for tendonitis was noted (*P* < 0.04) with treated horses being more lame on pressure induced lameness at days 7 and 14. Average goniometeric measurement for pain-free range of motion in OA horses showed no significant difference between treatment groups or time (Table 3).

### Lameness Scores

Lameness scores and lameness index (Table 4) showed that clinical trial horses were significantly improved with dental pulp tissue particle treatment compared to control horses (*P* < 0.013). Post-test analysis showed no significance difference between groups before injection at day 0, but a lower lameness score after injection in the treated group for day 7 and day 14 that was not seen in the control group (*P* < 0.007, *P* < 0.009 respectively). The treated group had a significantly greater number of horses that improved and with a sound gait at days 7 (*P* < 0.014) and 14 (*P* < 0.02) compared to control horses. The odds that treated horses would respond were 9-fold (day 7) and 12.7-fold (day 14) more likely than control horses. Lameness grades in the ST horses were significantly lower in the treated compared to control horses (*P* < 0.0005) with the tendonitis horses showing a stronger significant difference (*P* < 0.001) than the desmitis (*P* < 0.01) horses. Lameness scores within the OA horses improved, although not statistically significant, with treatment (*P* < 0.07) and ST horses showed greater improvement than OA horses (*P* < 0.001).

**Table 4:** **Lameness scores and indices (mean ± SD) to assess pain after injection of dental pulp tissue particles or carrier vehicle in horses with distal limb lameness due to osteoarthritis (OA) or soft tissue (ST) injury of tendonitis or suspensory desmitis**.

Parameter	Disease	Group	Day 0	Day 2	Day 4	Day 7	Day 10	Day 14
Lameness 0–5	All	C	3.3 ± 0.7			3.3 ± 0.7		3.4 ± 0.5
		Tx^+^	3.4 ± 0.8^a^			2.7 ± 0.7^b,^^		2.9 ± 0.6^b,^*
	OA	C	3.6 ± 0.6			3.7 ± 0.6		3.5 ± 0.6
		Tx	3.9 ± 0.5			3.5 ± 0.4		3.7 ± 0.4
	ST	C	3.0 ± 0.4			2.9 ± 0.6		3.1 ± 0.1
		Tx^+^	2.9 ± 0.6^a^			1.9 ± 0.1^b^		2.1 ± 0.1^b,^*
	Suspensory	C	3.0 ± 0.5			2.8 ± 0.8		3.0 ± 0.1
		Tx^+^	3.0 ± 0.5^a^			2.2 ± 0.6^b^		2.5 ± 0.9^a,b^
	Tendon	C	3.0 ± 0			3.0 ± 0		3.0 ± 0
		Tx^+^	2.3 ± 0.5^a^			1.3 ± 1.0^b^		1.0 ± 1.1^b^
Lameness severity score (0.1–0.9)	All	C	0.4 ± 0.2			0.18 ± 0.3		0.2 ± 0.3
		Tx	0.5 ± 0.3			0.4 ± 0.3		0.4 ± 0.3
	OA	C	0.4 ± 0.3			0.4 ± 0.3		0.3 ± 0.2
		Tx	0.5 ± 0.3			0.6 ± 0.3		0.6 ± 0.3
	ST	C	0.5 ± 0.2			0.4 ± 0.2		0.5 ± 0.1
		Tx	0.4 ± 0.2			0.2 ± 0.3		0.2 ± 0.3
Lameness response to flexion/pressure (0–5)	All	C	3.8 ± 1.0			3.7 ± 1.5		3.5 ± 1.5
		Tx	3.8 ± 1.0			3.0 ± 1.7		3.0 ± 1.6
	OA (to flexion)	C	3.9 ± 0.9			4.2 ± 2.0		3.5 ± 1.8
		Tx	4.4 ± 0.7			4.0 ± 1.5		4.0 ± 1.5
	ST (to pressure)	C	3.5 ± 0.8			3.4 ± 0.9		3.5 ± 0.8
		Tx	3.2 ± 0.9			2.2 ± 1.7		2.2 ± 1.5
Lameness index	All	C	7.3 ± 1.4			7.3 ± 1.9		7.0 ± 1.3
		Tx	7.6 ± 2.8			6.1 ± 3.18		6.3 ± 2.9
	OA	C	7.5 ± 1.7			8.0 ± 2.0		6.8 ± 1.8
		Tx	8.8 ± 1.2			8.1 ± 1.3		8.2 ± 1.2
	ST	C	6.98 ± 1.0			6.6 ± 1.6		7.0 ± 1.0
		Tx	6.4 ± 1.5			4.3 ± 3.4		4.5 ± 1.98

*Lameness index is the sum of the lameness score, the lameness severity score, and the lameness score after joint flexion (OA) or pressure (ST). Total possible score 10.9. Significance only denoted for lameness parameter*.

*P < 0.05 between control and treated group.

*^+^P < 0.04 differs from baseline within group*.

*^^^*P* < 0.07 between control and treated group*.

*P < 0.01 different letter superscripts differ from baseline within group*.

### Kinetic Gait Analysis

Kinetic gait analysis was complicated by horses with moderate to marked lameness (grades 4 and 5) that did not permit collection of all data at the predetermined velocities with four horses recorded at lower velocity (two control horses recorded at 2.0–3.0 m/s and two treated horses recorded at 0.5–1.5 or 1.5–2.5 m/s, respectively). A slower velocity would appear as more lame (lower VFP) and would incorrectly appear as a lack of response to treatment or an underestimation of improvement. Regardless, velocity did not significantly differ between groups or across trials. AI-VFP and CV-VFP decreased with time and had interactions with treatment and disease. AI-VFP and CV-VFP decreased compared to baseline in pulp-treated horses (*P* ≤ 0.05), particularly within treated OA horses, both corresponding to less lameness. Pulp-treated horses started with a mean of 11% AI-VPF and at day 14 and ended with a mean of 6% AI-VPF, an almost 2-fold improvement toward soundness. AI-VFI in OA (*P* < 0.04) horses and CV-VPI (*P* < 0.03) was lower in the pulp-treated horses compared to baseline that was not seen in the control horses, both corresponding to less lameness. When ST horses (*n* = 10 treated and *n* = 10 control) were evaluated as a subset of all horses, the CV-VFP was significantly decreased in treated horses at day 14 compared to control horses at day 14 and tended to be lower than baseline, indicating less lameness. Pulp-treated horses had CV-VPF transition from a mean of 6% CV at baseline to a mean of 3.5% CV at 14 days, again a similar ~2-fold improvement corresponding to improvements in lameness. For the sound contralateral limbs in these horses, CV-VFP was a mean of 6.4 at baseline and remained at 6.8% CV at day 14 showing no change. These parameters correlated with the significant improvement of lameness scores for this subset of treated horses (*P* < 0.007).

### Client Questionnaires

**Figure 6:**
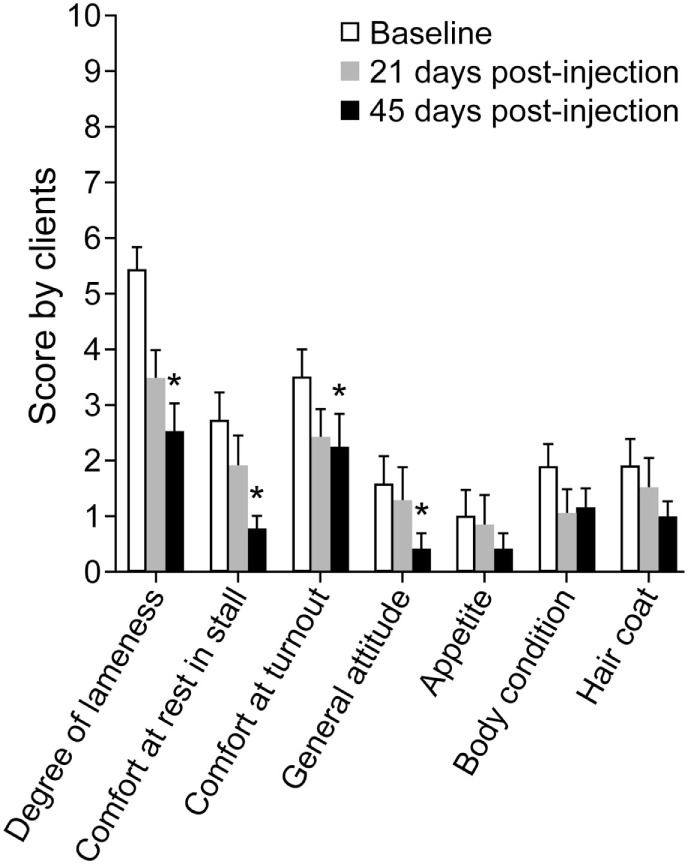
**Graph of mean ± SEM client survey scores for assessment of lameness and discomfort before (Baseline), 21 days post-injection, and 45 days post-injection of dental pulp tissue particles**. Client scores decreased across time (*P* < 0.001), significantly in four categories (lameness, comfort in stall, comfort in turnout, and general attitude) by day 45.

Client scores decreased (corresponding to improvement) across time (*P* < 0.001), significantly in four categories (lameness, comfort in stall, comfort in turnout, and general attitude) by day 45 (Figure 6). At long-term follow-up, >2.5 years after injection, 20 clients responded to the questionnaire which corresponded to a 50% response rate. Of the 20 responses, four horses had died or were euthanized for reasons unrelated to the injection or OA/ST injury. Of the 16 responding clients with live horses, 12 commented on the use of the horse and of the 12, 10 horses (83%) were being ridden (two at national competition level). The two horses not being ridden were at pasture, one as a brood mare and one retired. Seven of the 10 horses in work were sound (score 0).

### Owner Selection for Additional Treatment

All horse owners in the control group opted to receive the dental pulp tissue particle injection after the termination of the study. Twelve of the 20 horse owners in the treated group opted for at least one more injection after the end of the data collection phase at 45 days and two horses received three injections. No adverse events were noted in these horses and no horses worsened in lameness.

## Discussion

This prospective, randomized, blinded, and controlled clinical trial is the first to show that viable dental pulp tissue particles were effective to improve lameness when injected into an OA joint or injured tendon or ligament compared to a placebo-control solution within 1 week that sustained for at least 2 weeks. The client assessment survey for lameness and other parameters of comfort showed significant improvement from baseline that was sustained for at least 45 days supporting the findings from the controlled clinical trial. Clients were satisfied with the level of comfort of their horse and the injection. Clients reported that no horses during these 45 days had a life-threatening event or worsening of the lameness. Long-term follow-up (range 2.5–3 years) had many horses sound and in use without adverse events or worsening of the condition or lameness. Interestingly, horses with ST injury improved in lameness to a greater degree than OA horses. It is reasonable that the dental pulp tissue particles were more effective in ST injury possibly due to similarity of connective tissue phenotype or possibly ST injuries heal better than degenerative cartilage in OA. ST injury horses may also have had less chronic and less permanent injury than OA horses. All horses had to be lame for >1 month to meet inclusion criteria, but OA horses had to have visible changes on a radiograph that likely required the disease to be ongoing for longer than 1 month. In the case of ST injury, it would have been possible for the injury to be just over the 30-day criteria and still meet the lameness and ultrasound criteria. Further study correlating duration of ST injury, presence of fibrosis, and size of lesion with outcome is warranted to further define the treatment recommendations. Another explanation for less response by OA horses is that a hallmark of OA is cartilage damage and many of these horses may have had permanent injury that is beyond pain relief or restoration at least within the time frame of this study.

Our synovial fluid data demonstrated lack of persistent inflammation in the joint fluid by 2 weeks after injection. This was anticipated based on our preliminary data in which the inflammatory response of the joint was transient and returned to normal even in OA joints by day 7 (Figure 2). Importantly, however, there was a persistent (at least to 2 weeks) significant decrease in two key inflammatory cytokine interleukins in the treated group that was actually increased in the control group. This anti-inflammatory effect of dental pulp tissue particles in OA joints may provide a longer term influence that could benefit cartilage at a later time point than was studied here. To have been able to evaluate the healing or regeneration of cartilage in OA, or ST, horses, would have required an 8–12 months controlled experimental model study or clinical trial that is not only costly but often requires invasive surgery, costly MRI, or euthanasia to obtain tissue samples for quantification of results. Well controlled clinical trials, as performed here, offer the advantage of not only determining efficacy of a treatment but also effectiveness in natural occurring disease states that have comorbidities and expectations of return to work for this cohort. Importantly, our study focused on proving effectiveness of dental pulp tissue particles as a treatment to improve lameness using these three target diseases that have been shown to respond to MSC in many other studies as summarized in Section “Introduction.” To accomplish this goal, only 2 weeks of the prospective, blinded, randomized, and placebo-controlled clinical trial with quantitative metrics was needed. Client assessments of lameness and comfort in a prospective study using a validated survey also supported improvement in discomfort and lameness from baseline out to 45 days. No horses were able to work prior to enrollment, no horses worsened in ST injury or progressed in OA as measured in the short term by ultrasound or radiograph, respectively. Of the clients that commented on current use of the horse in the final questionnaire at >2.5 years, 83% commented their horses were in work and being ridden. In concert, these data support a pain-relieving effect lasting longer than most conventional pain medications without repeated dosing and an anti-inflammatory biomarker change in the joint fluid of OA horses at 2 weeks supporting a biologic improvement in joint environment.

The design of this study represented a hybrid clinical trial with a primary outcome of pain relief. Client-owned animals with natural occurring clinical disease were kept in the hospital for quantitative assessments to control for many factors known to affect lameness, such as exercise, stall size, and evaluators. A team of qualified and trained handlers and evaluators, blinded to treatment, were used for the lameness examinations, physical examinations, and subjective semi-quantitative scoring of edema, pain on pressure, pain on flexion, or pain-free range of joint motion. Our study design permitted the use of fewer animals than a typical field trial (51) in which hundreds of horses often participate. In our study, clients evaluated relevant parameters that did not duplicate our assessments of lameness, but were additive. Horses were evaluated in the home environment and owners co-signed that their horses were maintained in the same environment situations as the baseline assessments providing other controls that strengthen these client assessments over typical field trials. Our reports using similar hybrid models of OA in horses (22) have confirmed that the use of these hybrid clinical trials has reduced the coefficient of variance for the force plate, for example to between 6 and 8% (52, 53), an acknowledged low animal variability, even for experimental animal models of lameness. These studies also offer the option for a client assessment study with the collection of data on client opinion and perception of their animal improvement which can give an estimate of the public reception to the treatment. In dogs with OA, similar standardized client questionnaires (21, 23) have been used to observe the well accepted measures of pain at home, such as climbing stairs, jumping into the car or on the couch, getting up for treats, or circling before bedding. These instrument surveys capture these subjective but valid assessments at home. The first four categories in our survey have been validated to correlate to lameness scores on the same animal and lameness on the force plate (22) (see text footnote 16). Our goal with the client assessment was to capture other more subjective assessments that could represent discomfort. Indeed, these first four categories were statistically significantly improved in the 21- and 45-day assessment. Post-analysis of data showed that random case assignment did not inadvertently bias this study for front versus hind lameness, right versus left limb affected, cause of disease, severity of baseline lameness, gender, age, or breed.

Use of allogeneic intra-articular cells in horses has met with mixed recommendations. An earlier paper published a statistically insignificant, but increased inflammatory response to allogeneic compared to autologous placenta-derived MSC that was less with half-siblings (11). Recent papers reported lack of induced lameness or adverse reactions following injection of MSC into cartilage defects or OA in stifle joints of horses (27, 28, 30). Allogeneic bone marrow-derived cultured MSC injected as 5 × 10^7^/mL into fetlock joints caused a greater peak transient inflammatory response than autologous cells, but less than xenogeneic cells (4). Only xenogeneic cells invoked immune activation as measured by number of CD8+ T cells or inflammatory cytokine production when injected MSC were cultured back with the horse’s peripheral blood monocytes taken 120 days after original MSC intra-articular injection (19). In concert, these data suggested that an immune or inflammatory reaction to allogeneic cells is either lowly detectable or undetectable, without recognizable clinical effects, and for at least 120 days. Our data from this study would corroborate these findings as a mild and transient inflammation. Further safety studies looking at the immune-tolerance of the allogeneic pulp are warranted. The advantages of the use of allogeneic cells are many, most importantly an “off the shelf” access and full characterization of the product. In the case of this dental pulp tissue particle product, characterized as containing CD44, CD90, and CD105 positive cells reflecting a mesenchymal progenitor as well as CD34 and CD45 hematopoetic progenitor profile, can be available within 24 h of order for injection in practice.

The current study had minimal inflammation in the acute phase after injection that was transient in both groups and resolved rapidly. We reported similar findings for autologous protein solution (22). The inflammation was mild and focal around the injection site and, as expected, was greater in the pulp-treated group. We elected to not treat these horses with anti-inflammatory medications or a pressure bandage so as not to interfere with our outcome assessments. These recommendations, that are standard in practice, would be expected to completely eliminate these signs. Although non-steroidal anti-inflammatory drugs are often withheld after platelet concentrate injections so as to not suppress platelet activation and release of growth factors, to the authors’ knowledge, there is no known interaction of non-steroidal anti-inflammatory drugs and dental pulp tissue particles. Importantly, there were no adverse events or worsening of lameness at any time point in the study.

This study did have some limitations. Our study was designed to prove that dental pulp tissue particles could reduce lameness and pain in natural occurring disease conditions in horses. Our study was not designed to prove the duration of the effectiveness nor the regenerative or healing potential of these particles. A different design and longer term controls would have been necessary to prove the duration. Although our study did supply client assessment studies that support an effect to at least 45 days compared to the baseline this is a lower level of evidence due to lack of matched untreated controls or vehicle controls for the same time period. These follow-up assessments also provide evidence that the product, administered at this clinical dose, was not visibly harmful to the horse or the condition out to 45 days (>6 weeks) based on the client assessment. Our study does not determine if other longer term changes in the disease or tissues could be associated with injection. A full safety study of the product would likely include study at doses higher than recommended clinically. Another limitation of any biologic study is the unknown metric to guide dosing. Biologic products in horses are not currently dosed on body weight or any recommendations based on lesion size or severity. Our selection of a single dose for all horses may have contributed to variability in our findings, but this guiding information is not currently available.

In our study, the force plate data corroborated our lameness scores with statistically significant correlations and shift in AI-VFP and CV-VFP in the direction that corresponded to greater loading of the limb and less variability step-to-step (22, 52, 53). The AI-VFP parameter is used preferentially in studies in which both forelimb and hindlimb lameness are included since AI-VFP measures the normalized difference in the VFP between the lame limb and the contralateral limb (22). If a horse is changing from lameness toward soundness, the difference between the lame and contralateral limb will approach 0. Variability in limb loading step-to-step (CV-VPF) has been another parameter that has directly correlated to degree of lameness in other studies (52, 53). Horses with lameness place load on the lame limbs inconsistently, producing greater variability (CV) within the five valid trials recorded in these studies. Pulp-treated horses significantly reduced CV-VFP from a mean of 6–3.5%, not seen in the controls. Overall, despite some limitations in the force plate data collection, the findings correlated highly with our lameness scores and supported our findings of a reduction in lameness in horses with OA, tendonitis, or desmitis that were treated with dental pulp tissue particles.

In summary, a single intra-articular dose of 10 × 10^6^ dental pulp tissue particles in 2 mL of sterile isotonic buffered saline solution significantly improved lameness in horses with OA or ST equine injuries compared to the same solution without dental pulp tissue particles. Minimal transient edema, greater than control, was localized to the injection site out to day 7. Dental pulp tissue particles had significantly lower inflammatory mediators in the OA synovial fluid after treatment that actually increased in the controls, and significantly improved client scores for lameness and comfort at 21 and 45 days. Of the 12 clients that commented on current use of the horse in the final questionnaire at >2.5 years, 10 commented their horses were in work and being ridden. In conclusion, use of dental pulp tissue particles was effective in the treatment of pain and lameness due to OA, tendonitis, or desmitis, and represents an injectable therapeutic choice that has regenerative potential.

## Ethics Statement

The study was approved by the University Laboratory Animal Care and Use Committee, the Veterinary Medical Center Council Research Advisory Committee, the Veterinary Medical Center Board for VMC, and the OSU CVM Clinical Trials Office Review.

## Author Contributions

AB designed, performed inclusion criteria assessments, signed all consent forms and paperwork, managed clinical trial office (OSU) paperwork, assigned scores for lameness data, managed and overseen all aspects of the study, and wrote the manuscript. NR, study manager, performed much of the day-to-day management of collection of force plate data, maintenance of blinding. MK assisted in all force plate work, studied force plate data, and analyzed extensively FP data. AK, direct assistant, oversaw blood draws, following of GCP protocol for blood processing, handling of stem cells and paperwork, and served as the unblinded coordinator of materials in a GCP manner. Performed QA on data entry and performed statistics with AB and our statistician. NS and RL, veterinarians to perform, collect, and manage all collection of physical examinations (over 300) and client contacts regarding options for treatments, data entry for all blood work and exams, data statistics, and tabulation of physical exams, and assisted with force plate, lameness exams, and scoring paperwork. JS performed U/S collection and data analysis. RK performed all elizas and snyovial fluid assessments, LZ scored all rads and oversaw US and data management, MB recruited cases and backup all lameness exams.

## Conflict of Interest Statement

The author ALB served as a consultant for a different but related company investigating human application of dental pulp to osteoarthritis in people. The remaining authors declare that the research was conducted in the absence of any commercial or financial relationships that could be construed as a potential conflict of interest.

## Footnotes

^1^Post-market study of articular cartilage defects of the knee treated with *DeNovo* NT, Natural Tissue Graft NCT00791245. Zimmer Orthobiologics May 3, 2016. https://clinicaltrials.gov/ct2/show/NCT00791245?term=carilgae+repair&rank=11.

^2^Randomized controlled trial to evaluate the efficacy of a neocartilage implant in the management of ICRS grades 3–4 articular cartilage lesions of the knee (Neocartilage Implant/*DeNovo*®ET Engineered Tissue Graft) May 3, 2016. https://clinicaltrials.gov/ct2/show/NCT01400607?term=cartilage+repair&rank=12.

^3^MarrowStem® and BioCUE™ BBMA (blood and bone marrow aspirate). www.biomet.com.

^4^EStem®, Pall Corporation. www.pall.com.

^5^Harvest® Bone Marrow Aspirate Concentrate (BMAC®) System. www.harvesttech.com.

^6^InGeneron Incorporated, ARC™ (adipose-derived regenerative cells) System. www.ingeneron.com.

^7^VetCell Therapeutics, ReGen OA (adipose-derived regenerative cells). http://vetcelltherapeutics.com.

^8^Cell-based products for animal use. Guidance for Industry #218. Center for Veterinary Medicine (HFV-114), Food and Drug Administration. http://www.fda.gov/AnimalVeterinary/default.htm or http://www.regulations.gov.

^9^Pulpcyte® StemLutions (currently VetGraft) Columbus, OH, USA. www.VetGraft.com.

^10^Sterile Isotonic Saline Solution (manufactured under ISO 9001 and 13485), Preservation Solutions, Elkhorn, WI, USA.

^11^eBiosciences. www.ebioscience.com.

^12^Becton Dickinson LSR-II flow cytometer.

^13^Thermo LTQ_XL Orbitrap, Bruker Maxis. https://cancer.osu.edu/research-and-education/shared-resources/proteomics/services.

^14^www.ncbi.nlm.nih.gov.

^15^Stat Trek. Available at: http://stattrek.com/statistics/random-number-generator.aspx. Accessed Jan 6, 2016.

^16^Prospective, randomized, blinded, and controlled clinical trial (Bertone PI) to study reduction in pain and lameness by opioids for distal limb pain in horses, data unpublished, 2014.

^17^Standards for Tissue Banking, the American Association of Tissue Banks (February 29, 2012—13th edition) McLean, VA, USA. www.atb.org.

^18^Clinical Pathology Laboratory, The Ohio State University, Columbus, OH, USA.

^19^R&D Systems Inc., Minneapolis, MN, USA.

^20^Sato I, Uppsala, Sweden.

^21^Kistler Instrument Corp., Amherst, NY, USA.

^22^eFilm Workstation® 4.0.3, Merge Healthcare, http://estore.merge.com, Academic License, 2016.

^23^SAS, version 9.2, SAS Institute Inc., Cary, NC, USA.

^24^STATA, version 11, Stata Corp LP, College Station, TX, USA.

^25^http://Statisticalsolutions.net.

^26^Merck Manual Veterinary, Merck & Co., www.merckvetmanual.com, 2015.

## Abbreviations

AAEP, American Association of Equine Practitioners; CBC, complete blood count; DDF, deep digital flexor tendon; GMP, good manufacturing processing; IL-1ra, interleukin-1 receptor antagonist; IL-1β, interleukin-1β; IL-6, interleukin 6; IL-10, interleukin 10; OA, osteoarthritis; PRP, platelet-rich plasma; SDF, superficial digital flexor tendon; ST, soft tissue; WBC, white blood cell.
